# Impact of Food Waste on Society, Specifically at Retail and Foodservice Levels in Developed and Developing Countries

**DOI:** 10.3390/foods13132098

**Published:** 2024-07-01

**Authors:** Ewen Cameron David Todd, Dima Faour-Klingbeil

**Affiliations:** 1 Ewen Todd Consulting LLC, Okemos, MI 48864, USA; 2 DFK for Safe Food Environment, 30559 Hannover, Germany; dima.faour@gmail.com

**Keywords:** food waste, food loss, retail sector, foodservice sector, sustainability, food safety, food waste mitigation

## Abstract

Food loss and waste pose significant challenges in both industrial and agricultural food production sectors. In recent decades, their environmental and economic impacts have intensified due to increasing food demand, heightened production activities, and varying standards across the global supply chain. Specifically, the complexities surrounding the causes of food waste at the retail and household levels persist as a multifaceted issue, constituting a crucial topic in food policy. This is driven by various interplaying aspects, such as food security, safety, quality, and environmental sustainability, including greenhouse gas emissions from decaying food, water pollution from leaching, and the proliferation of landfills. Additionally, consumer concerns and financial losses exacerbate the urgency of addressing this issue. Therefore, this paper aims to highlight these complexities as a focal point of its discussion by the examination of interconnected causes of food waste and potential solutions and initiatives to reduce food waste occurring at these critical points in the food supply chain. Less attention has been paid to retail and foodservice than production and consumer sectors, and this review specifically focuses on these industries, where food waste is more important than food loss. This review also looks at examples in developing countries which have received less attention until now. We believe that because of the complexity of the process to reduce food waste across the food supply chain, and the many stakeholders involved, the goal of a 50% reduction by 2030 set by the United Nations will be difficult to achieve on time.

## 1. Introduction

Food loss and waste reduction has become an urgent necessity globally to combat hunger, malnutrition, resource depletion, and global warming. Around 691 to 783 million people suffered from hunger in 2022, with lost and wasted food contributing to 38% of the total energy usage in the global food system [1]. It is estimated that 13% of the food produced globally is lost between harvest and retail, and another 17% is wasted in retail and at the consumption level [2]. The FAO [3] now has the largest online global collection of data on both food loss and food waste and their reported causes.

**Figure 1:**
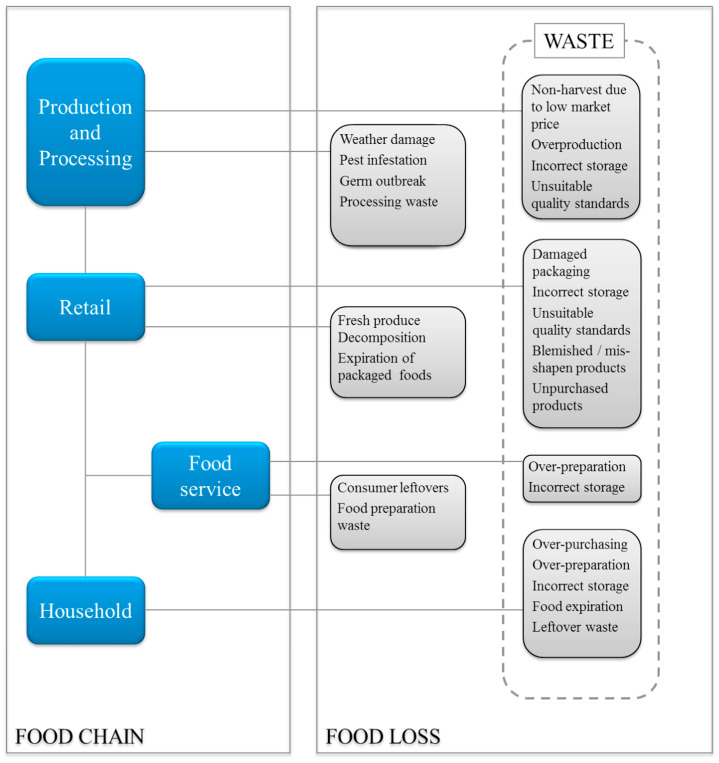
A conceptual model of food losses, including waste generated all along the food chain. The figure was reproduced from Cicatiello et al. [4] with permission from Elsevier, Amsterdam, The Netherlands, 2016.

Food loss and food waste are separate items. According to the FAO, food loss is “the decrease in quantity or quality of food and are the agricultural or fisheries products intended for human consumption that are ultimately not eaten by people or that have incurred a reduction in quality reflected in their nutritional value, economic value or food safety”. Food waste, which is a large contributor to food loss, is “the discarding or alternative (nonfood) use of food that was fit for human consumption—by choice or after the food has been left to spoil or expire as a result of negligence”. Slightly different definitions come from Cicatiello et al. [4], who state the following: “Food loss can be defined as a qualitative or quantitative drop in the food supply due to a reduced nutrient value of the food or to a decrease in its weight or volume. When an edible item is lost due to these processes, food waste occurs. From this perspective, food waste only concerns edible items, and it is directly linked to human action or inaction. Food waste can therefore, be conceived as the result of decisions made by consumers, supply chain actors or other stakeholders, and it represents a subset of the total food losses”. To summarize, food loss occurs at the farm level, during the production and post-harvest stages, and during distribution in the value chain before it reaches the consumer and is usually discarded despite its suitability for human consumption. Food waste is also caused by spoilage and results from poor planning regarding food preparation, storage, reuse, or management [5]. If we consider that food waste consists of both avoidable and unavoidable waste [6], examples of edible avoidable waste are bakery products, fruit, vegetables, and meat, and possibly edible avoidable waste products include bread crusts, potato skins, and apple cores. Inedible unavoidable food waste is created during food preparation and includes bones, fat, eggshells, nut shells, and pineapple and citrus fruit skins. Figure 1 illustrates the different causes of food loss and food waste. Some studies have categorized more of the loss items as food waste, such as the expiration of packaged foods, food preparation waste, and consumer leftovers at restaurants. As later discussed, different definitions and measurements of edible and inedible waste confuse policy planners when obtaining a baseline level of total waste for developing reduction goals and strategies. For instance, not all studies separate loss from waste and combine them as food loss and waste (FLW), perhaps because of the difficulty of obtaining clear definitions of both.

Food loss starts at the farm level not only because of vagaries of agriculture and animal production because of the weather, disease, spoilage, labor shortages, and changeable consumer demand, but also because of variable market prices that can force farmers to limit the amount of food that is harvested compared to what was produced. Degradation in food quality and microbial spoilage are also responsible for the loss of approximately 25% of all food produced globally [7]. But a large part of this loss also occurs at the retail, foodservice, and consumer stages in industrialized countries. For instance, shipping food to processing plants may also cause losses through spoilage or imperfect-looking items. Processing fruits and vegetables will result in trimming (line waste), even though much may still be used as edible food items, e.g., potato peels. Also, during processing, production line changes and regular cleaning schedules will cause some product waste.

The impact of food waste is widespread globally; for example, up to 40% of the US food supply is wasted annually. This is equivalent to 549 pounds of food waste per person that was sent for disposal in 2021 alone. This means that millions of pounds of wasted food end up in landfills each year with an estimated total of 91 million tons in 2021. Even in developed countries, large numbers of people live in extreme poverty because they are economically unable to benefit from a sufficient food supply. This is in addition to a loss of food along the food chain through human production, transportation and marketing operations, and the eating habits of consumers. Cicatiello et al. [4] note that sustainability in retail can increase food waste. Instead of nourishing people, this discarded food leads to further waste, including unnecessary water, labor, and energy usage throughout the food production, processing, transportation, preparation, storage, and disposal processes. Unfortunately, the reported figures on food waste may not accurately capture the full extent of food waste. Many retail companies classify unsold products as expenses within their data systems. Since unsold products result in lost sales or turnover, information regarding food waste often remains hidden within cost accounting practices and is typically not included in food loss data; non-sold products are often recorded as financial losses [8]. However, the retail stage accounts for smaller portions of loss and waste in comparison to other stages in the food supply chain. Nevertheless, its actions significantly impact how food is managed both by suppliers and users in the food chain. By controlling the distribution of food items, retailers influence consumers’ purchasing habits [8]. Only by understanding the root causes of loss and waste can attempts be made to reduce these. From what we know, the causes are complex, with food products being impacted by food safety regulations, industry guidelines, climate change, consumer preferences, and charities interested in receiving donations of blemished or close-to-expired products [9]. The same applies to household food waste, especially with regard to how food retailers influence and interact with household food management practices. Thus, this paper sheds light on both food loss and food waste, but more specifically, it explores the interconnected causes and potential solutions for food waste occurring at two crucial points in the food supply chain: retailers and hospitality. This is an important topic in food policy because of the interplay of various aspects, such as food security, food safety, food quality, environmental damage, greenhouse gasses (GHGs) from decaying food, water pollution from leaching and expanding landfills, as well as consumer concerns and financial loss [4].

## 2. An Overview of the Overall Burden of Food Loss and Waste

Globally, the loss of food can be as high as 30% of cereals, 40–50% of fruits and vegetables, including root crops, 20% of oil seeds and meat and dairy products, and 35% of fish, which is caused by the poor harvesting, storage, transportation, processing, packaging, and marketing of these products, often as a result of compromise in the cold chain, particularly in low-income countries. In medium- and high-income countries, food waste is more likely to occur at the retail level and through consumer behavior, restrictive government policies, and concerns over loss of food quality and risks to food safety [10]. In both Canada and the EU, retail food waste contributes a significant percentage to the overall food waste along the food supply chain, at 12% and approximately 5%, respectively [11]. These foods encompass all the resources used and environmental impacts generated upstream in food production, processing, and packaging. It is likely that much of this food could still be safe for human consumption if food item inventories at the retail level are carefully designed and followed and if consumer plans for appropriate purchase and storage practices are adhered to. Nevertheless, food safety aspects should have priority in food waste prevention programs since popular food waste mitigation initiatives could compromise that safety [12].

According to ReFED [13], surplus food cost USD 473 billion (B) in the United States (2022 data). Of this, 90.5%, costing USD 428 billion to the economy, involved food waste compared with recycled food (7.6%) and donated food (1.9%). While the financial cost of uneaten food is the greatest for consumers, there was a food surplus across the whole food supply chain, costing USD 246.7 billion in total (farms: 16.8%, USD 12B; manufacturing: 14.7%, USD 40.3B; consumer-facing businesses: 20.2%, USD 169.2B; homes: 48.2%, USD 25.2B). From this assessment, consumer-facing businesses are the most important contributors to FLW. These businesses include retailers (28%, USD 31.2B), full-service restaurants (34%, USD 67.6B); limited-service restaurants (14%, USD 25.3B); and other foodservice establishments, e.g., health care, assisted living, and the military (44%, USD 48.7B). Most of the surplus food comes from perishable items, as to be expected, including the following: produce (35.3%), prepared foods (20.3%), dairy and eggs (16%), dry goods (12%), fresh meat and seafood (5.7%), frozen foods (4.9%), ready-to-drink beverages (3.4%), and breads and bakery items (2.4%).

Discarding food that is still edible due to confusion over best before and use-by dates is a significant problem at both the retail and consumer levels. Charlebois et al. [14] discuss the history of food safety and “best before” dates and the current use of these food labeling practices in Canada, the United States, and the United Kingdom (UK). Food label dates in Canada are determined by the industry as a quality indicator and are not thresholds for food safety, as many purchasers believe, and some food is unnecessarily discarded, contributing to GHG emissions. As a result, about 10% of food waste in the European Union (EU) is linked to date marking.

The literature on food waste has so far focused on the quantification of the total food losses along the supply chain [15,16,17] to highlight the negative implications of this phenomenon as well as its impact on the food system as a whole [18]. It is difficult to determine how much retailing contributes to wasted food; the European Commission estimates that this amount is only 5%, but this is considered an underestimate by Cerciello et al. [19]. For instance, over 30 tonnes of food of animal and nonanimal origin, in addition to 210 eggs and 606 L of soft drinks, confiscated from production sites, warehouses, stores, and foodservice establishments, were destroyed by the Bulgarian Food Safety Agency due to inappropriate storage, incorrect labeling, or use-by dates [20].

In the United States, food waste is estimated to take up between 30 and 40% of the food supply. This estimate, which is based on estimates from the USDA’s Economic Research Service of 31% of food loss at the retail and consumer levels, corresponded to approximately 133 billion pounds and USD 161 billion worth of food in 2010 [21].

## 3. The Causes of Food Waste at the Retail and Hospitality Levels

### At the Retail Level

The food waste in retail is multifaceted and can be caused by various factors, which should be addressed for waste mitigation. Moraes et al. [9] discussed the causes of food waste and its disposal, and they determined that it is very complex as procedures for handling such waste are not only impacted by governments and consumers, but also by the environmental impact, internal codes of practice in industry, and organizations interested in receiving food donations.

Schneider and Eriksson [8] referred food waste factors to three main stages at the retail level:

(1) Pre-storing food waste induced by retail: At this stage, retailers have a significant influence on quality standards, particularly for fresh produce, and individual marketing standards play a crucial role in determining losses at the global supplier level. Imperfect products typically do not make it to the retail shelves of international or national chains. When products fail to meet the specifications outlined by the purchasing department of these retail chains, they are promptly rejected on-site. Return transportation costs often render it impractical to send them back. Instead, these products are frequently repurposed for uses such as animal feed, biogas production, or soil amendment. Additionally, take-back agreements and rejection policies sometimes place the burden of food waste on suppliers, allowing retailers to dispose of imperfect products without bearing the full cost. Additionally, the contracts for agreed amounts of delivered produce may contribute to systematic overproduction in agriculture as farmers seek to mitigate the risk of penalties for the inability to deliver food due to poor harvests.
(2) The in-store food waste stage: This stage does not account for food waste as the surplus may have already been repurposed for donation to social organizations or utilized as animal feed. However, it still constitutes an economic loss for retailers; operational issues leading to waste include an expired shelf life, visual defects, and overstocking due to inaccurate sales predictions.
(3) Post-store food waste induced by retail through in-store promotions like BOGOF (buy one, get one free): This stage contributes to household-level surpluses and subsequent waste when products reach their expiry dates.

Retailers, mainly in developed countries, often adopt stringent standards to safeguard their business reputation against food-related issues and to differentiate themselves in the market by meeting consumer expectations for quality [22]. However, there is a tendency to prioritize the appearance of products in these standards, which has been associated with significant levels of food wastage and the rejection of goods, many of which end up spoiling during distribution [23].

Grocery retailers depend on consumers’ buying options mainly for produce and packaged food products, especially perceived freshness. Because they are at the center of the supply chain, retailers can have a substantial influence on how food waste can be reduced. To keep up with consumer demand, retailers have to be concerned with both the quality and quantity regarding all of the different labels. Missteps create inventory issues, with the risk of some foods not being sold before their expiry dates. In 2022, retailers in the United States generated 4.99 M tons of surplus food, nearly 35% of which went to landfill or was incinerated as waste [13,24]. Most of this came from produce (31.3%), dairy and eggs (14.1%), and fresh meat and seafood (13.6%), and more than half was caused by concerns or confusion over freshness date labels. Although 19.5% was given as donations to food rescue organizations, any unnecessary waste leads to unnecessary economic and social costs for retailers and consumers alike.

Issues such as failures in maintaining the cold chain, noncompliance with food safety regulations or industry norms, and mislabeling by suppliers also contribute to this rejection of food at the retail level, resulting in subsequent waste [5]. Food safety, quality, and esthetic standards and guidelines set by the government and industry that would tend to increase waste could be amended to allow for retail food to be donated to charities with protection from legal action provided that their donations are deemed to be safe and made under sanitary conditions, even if they have expired. Pressure from shareholders and competitors may result in retailers promoting high-value products with an acceptable amount of waste offset by higher process. Choices made by shoppers determine the importance of the cosmetics of short-shelf-life products like fruits and vegetables; if those with blemishes are disregarded, this results in more food waste. Environmental issues affect the availability and shelf life of products, including the uncertainty of climate change with extreme weather events, which make inventories difficult to maximize for sales without waste. In the United States, an investigation on US retailers showed that 72.5% of meat and seafood items were categorized as losses attributed to spoilage and packaging damage [25].

Kafa and Jaegler [26] raised questions about how food loss and food waste (FLW) are defined and determined; nevertheless, they tried to compare studies using the different definitions. In their review of publications, these authors found that 38% used surveys as a direct method to measure FLW, 34% used modeling as an indirect method, and 6% of papers combined surveys and modeling; 5% combined surveys and weighing methods, and weighing alone was used in 4%. Other combinations accounted for ≤3%. In summary, surveys as a direct method and modeling as an indirect method were most often used to measure FLW in their review. However, the databases chosen may not reflect all of the possible sources of FLW, which would skew the results obtained. Actually weighing food waste as well as keeping diaries on the amount and types of FLW in a period of time, combined with keeping records of warehouse and sales data, may provide more accurate data, but these data are limited to operations that are willing to take the time and use resources to carry these measurements out. The authors consider that both direct and indirect methods should be used in country or multi-country studies, and more knowledge should be generated at the global scale, with upstream supply chain studies being more integrated with downstream supply chains with similar or comparable methodologies. For instance, they point out that if weight is the sole measure of waste, losing 1 kg of tomatoes in the field cannot be considered the same as losing 1 kg of tomatoes at retail in terms of nutritional or economic value. Methods at national levels to find out how much food is wasted vary as well as the effectiveness of strategies, if any, to reduce it.

The EU and the United States have tried to assess how well food waste is addressed and reduced along the food chain. Caldeira et al. [27] established a common methodology and minimum quality requirements for the uniform measurement of the level of food waste generated in EU Member States (MSs). The parameters used to evaluate them were as follows: the weight of food waste; waste composition analysis; surveys on attitudes, beliefs, and self-reported behaviors; diaries from daily records on the amount and type of food waste generated in a period of time; records, such as those from warehouse record books; evaluations of leftover food using visual methods; modeling using waste coefficients; inferring food waste by measuring inputs and outputs alongside changes in levels of stock including changes to the weight of food during processing; inferring food waste using data from companies or statistical agencies; and analyzing data from the literature. Some countries had more than one study, and four countries have not conducted any. Overall, not only were there few data on the amounts of food waste, but the MSs that conducted these studies used different food waste definitions and different quantification approaches, and whether or not inedible waste was included was not consistent across the studies. The focus of the study was the from farm to fork concept, but only data relating to retail and foodservices are given here. The amount of food waste generated at the retail and distribution levels ranged from 3 (Croatia) to 28–29 (Portugal and Germany) kg per capita. For restaurants and foodservices, the amount ranged from 6 (Croatia) to 31–32 (Belgium and Austria) kg per capita. They reported on a specific study conducted by Tesco showing 0.5% (UK), 1.2% (Ireland), and 1.2% (Czech Republic, Hungary, Poland, and Slovakia) of food waste as percentages of sales. The majority of the studies did not provide any type of information on the destinations of surplus food and food waste, but from the available data, most was valorized as animal feed, compost, or anaerobic digestion, and some was used for alcohol production. According to the World Resources Institute, a possible goal for a large grocery retailer would be to reduce waste, including inedible parts, that is sent to landfills by 50% by 2025 [28]; we have not heard that this goal is close to being achieved in 2024.

Food waste at the retail stage represents a significant economic issue given the low overall margins on food products and increasingly high operating costs, particularly at the store level, especially considering that a large proportion of the binned products are still edible. Retailers traditionally focus on minimizing costs, increasing profits, and providing excellent service for customers. Therefore, improved retail operations and higher on-shelf availability and fewer out-of-stock products for shoppers should lead to higher profitability at the store and retail organization levels. But this, by nature, would lead to over-stocking and more food waste, which Teller et al. [29] addressed. They sampled 28 stores from five organizations in Europe, divided into convenience stores, discount stores, supermarkets, and hypermarkets. They conducted semi-structured interviews with 12 food waste experts to confirm the findings of the case studies and simulations to show that the root causes of food waste are related to undesirable customer behavior and uncertain demand, inefficient store operations and stocking policies, and the high product quality requirements of both retail organizations and customers. But the root causes and their impacts differed across the type of store and what products were being carried. These were further influenced by the preferences of the shoppers, the policies of the corporate offices, and the operations of the stores themselves. The authors identified eleven root causes as follows: (1) a limited predictability of actual customer demand across what a store offers; (2) issues with employees, including insufficient staff who are knowledgeable and have experience, a low motivation to reduce waste, poor choices in ordering and replenishment, and a lack of managerial commitment; (3) products being delivered too close to expiry dates; (4) unnecessary high-quality standards set by corporate headquarters; (5) poor product quality due to the transport and delivery of perishable items; (6) customers’ pickiness regarding the appearance and quality of fresh produce; (7) too many products located at the store, especially during promotions; (8) corporate company requirements to facilitate 100% on-shelf availability of products; (9) a large number of product categories and too many choices within these; (10) secondary packaging units that are too large; and (11) problems related to forecasting and replenishment, especially with the fluctuating demand through marketing. Secondary packaging makes it possible to group products so that they can more easily be tracked in the store, but in supermarkets, it is too easy to overorder these, resulting in an excess of items in storage or on display that are unable to be sold. In hypermarkets, the root cause driving food waste is the high-quality standards of corporate offices, which can have products pulled from sale before expiry dates. The authors concluded that food waste occurs at the retail level due to the lack of coordination and understanding of the products by retail employees and how shoppers perceive the value and quality of the products offered, resulting in overstock. But suppliers for retailers also need to be included in a strategy to minimize retail food waste. The experience and commitment of managers in determining the right amount of product to be displayed is critical in any retail operation. Teller et al. [29] also stressed that the root causes may be different between different types of retail operations (hypermarkets, supermarkets, discount stores, and convenience stores) and that food waste is more extensive in larger operations that offer more choices and believe that products have to be of high quality and should be available at all times for retailers to keep attracting customers.

Colombo de Moraes et al. [9] conducted a comprehensive review of the causes of food waste in the retail industry and found 34 factors that contribute to food waste and 32 practices that could reduce the amount wasted. The authors stated that the information was theoretically assessed from articles published in five databases in the period from 2008 to 2017. Nevertheless, they demonstrated the complexity of the different aspects that contribute to food waste at the retail level. Many of these have already been documented, such as inventory issues, where a sudden change in orders can leave customers short or products unsold on the shelves (excess followed by a shortage); forecasting not only involves following the history of deliveries but also an awareness of weather and seasonal supplier issues, particularly in the time of climate change, which can disrupt crop growing patterns and delay shipping. Shoppers may also accept small blemishes of products during periods of high demand and low supplies, including imports, and reject them when local crops flood the markets. The use of radio-frequency identification (RFID) package tags and global positioning system (GPS) tracking can fine-tune traceability and product delivery timing if the cost allows. Temperature measuring devices can show if products have been kept at recommended temperatures during shipping; otherwise, the cold chain is broken. Inadequate packaging can result in damaged shipments that must be returned or sent to landfills. Improper storage at the warehouse or store level can lead to spoiled products or a shorter shelf life, as will unsuitable display cases or shelves. Colombo de Moraes et al. [9] consider it especially important for managers to be given flexibility for promotions, food bank donations, or the reuse of surplus food by secondary retail channels that sell food with lower-quality standards as long as the items are still safe to eat. However, in addition to being poor economical choices, improperly thought-out promotions may actually increase waste. But flexibility on the part of managers in the control of their supplies should not curtail information sharing within the chain, or regular communication with other retailers, to find ways to reduce waste. Corporate offices and store managers should set policies for managing food waste because confusing and unclear procedures for employees may cause an increase in waste. Partnering with food donation organizations can reduce what goes to landfills, and these partnerships should be started at the corporate level and operated at the local store level. Do retailers measure the amount of food that is wasted? There is no standard way of carrying this out, but unless records are kept by stores, there is no way to find out whether waste-saving devices are effective. The amount of food wasted will not change unless there is a managerial commitment to reduce it as much as possible and unless this policy is transmitted to staff through adequate knowledge and training to protect and preserve the items for sale as much as possible and avoid employee mishandling.

External stakeholders, such as legislators and their regulators, competitors, and consumers, affect the degree of food waste avoidance. For instance, there are laws and standards for producing food to be sold to the public, especially food safety and hygiene regulations, which force retailers to discard items prematurely if these are perceived as too restrictive, and any alternative uses of these foods are not considered economically or technically feasible. In contrast, some major stakeholders such as governments and consumer advocacy groups put pressure on food producers and retailers to meet their sustainability commitments. The authors summarize all of the issues they identified in Table 1 with causes and reduction strategies for food waste. The high number of possible causes for waste means achieving any substantial reduction will require much collaboration and persistence of stakeholders over decades rather than years.

These authors suggest that future research could involve (1) identifying and measuring the impacts of the causes related to external stakeholder groups (governments and regulators, competitors and industry entities, the environment, consumers, NGOs, and shareholders) and how retailers could be involved with possible solutions and (2) identifying how to change shopper buying patterns by measuring the impact of consumer awareness and strategizing education actions for waste reduction.

Some reduction practices are more achievable than others, and the authors admit that reducing food waste is a complex issue because it originates from a wide range of internal issues (which are potentially controllable at the retail chain or store) and external components (some of which are less uncontrollable or not controllable at all as they relate to the supply chain, consumer practices, and government oversight). Colombo de Moraes et al. [9] state that, because of the many stakeholders and components involved, a systematic and integrated management is required to adequately handle its complexity. How this is carried out is not explained. The large number of root causes and possible solutions listed in Table 1 show, however, how retailers alone can (1) develop procedures and policies related to quality, logistics, product display procedures, the management and measurement of waste, including edible and non-edible waste, and individual product types; (2) improve the coordination and collaboration of retailers with their suppliers; (3) promote the use of better technologies to improve precise demand forecasting and develop a consensus in retail waste measurement; (4) maintain appropriate targeted cold chains throughout supply and retail; (5) develop training and employee awareness regarding food waste avoidance practices; (6) use better protective packaging and more helpful product labels; (7) incorporate waste reduction into retail pricing and promotion policies; (8) develop standardized indicators and procedures for the measurement of food waste in retail, as well as food waste by suppliers, wholesalers, and producers; (9) improve food transport and display equipment to minimize food deterioration; (10) develop better waste management culture at the corporate or top management level of retail organizations through providing more awareness internally and engagement externally with stakeholders; (11) understand and improve the intrinsic quality of products marketed by retail, particularly with practices affecting products with short shelf lives; (12) identify and compare the peculiarities of the causes of food waste and reduction practices related to products from developing countries versus those from developed countries; (13) implement political measures where appropriate to amend and implement laws and regulations that promote and facilitate safe retail food donation and allow retailers to sell expired food that is still safe; and (14) encourage competitors and industry entities to establish more rigid trade and esthetic standards.

In 2016, the US Environmental Protection Agency (EPA) and the US Department of Agriculture (USDA, Washington, DC, USA) launched the US Food Loss and Waste 2030 Champions program to incentivize and recognize the efforts of organizations toward reducing FLW by 50% by the year 2030 [30]. In the United States, a substantial portion of FLW occurs at the stages of foodservice and homes within the food system. Apart from economic and nutritional losses, the environmental impact is also high with contributions to GHG emissions, the eutrophication of watersheds, the loss of land, and species diversity. For retail and foodservice operations, governments can develop education campaigns for consumers to understand date labels; educate potential food donors on donation liability laws; expand tax incentives for food donations by businesses; impose bans or fines for disposing of food waste in landfills; and provide incentives for redirecting food waste to other purposes for renewable energy credits. Companies should use cold-chain-certified carriers for transporting food; develop technologies to track the remaining shelf lives of food in retail inventory management systems; implement smart scales and technologies to track and record food waste during food preparation; and use apps to notify recipients of available excess food. Producers, including retailers and foodservice operations, can work with municipal authorities rather than directly sending waste to landfills to repurpose food waste through heat treatment, dehydration, and mixing for animal feed; use centralized anaerobic digestion for energy production; use commercial gray water aerobic digesters to break down discarded food; transport food waste to centralized composting facilities; and use water recovery facilities to convert food waste to biosolids for land application. Retailers can allow prepared foods to sell out near closing time without replenishing; discount older, slightly damaged items or items with a poorer cosmetic appearance and excess inventory; eliminate promotions that encourage the excessive purchase of repeat items; enable the purchase of smaller or customized portions (bulk bins and staffed delis); increase flexibility in contracting terms and grading standards for foods; redesign produce, deli, and seafood displays to use smaller containers; convert damaged products into prepared food items; divert excess processed food and unwanted produce to discount retailers; increase donations of unsold foods; divert trimmings and by-products to animal feed; and transport food waste to centralized composting facilities. At the foodservice level, staff can prepare smaller batches of food or cook to order, remove trays, and use smaller plates in buffet-style restaurants. At both the retail and foodservice levels, staff can be trained in and rewarded for waste reduction efforts such as optimal product handling and stock rotation.

**Figure 2:**
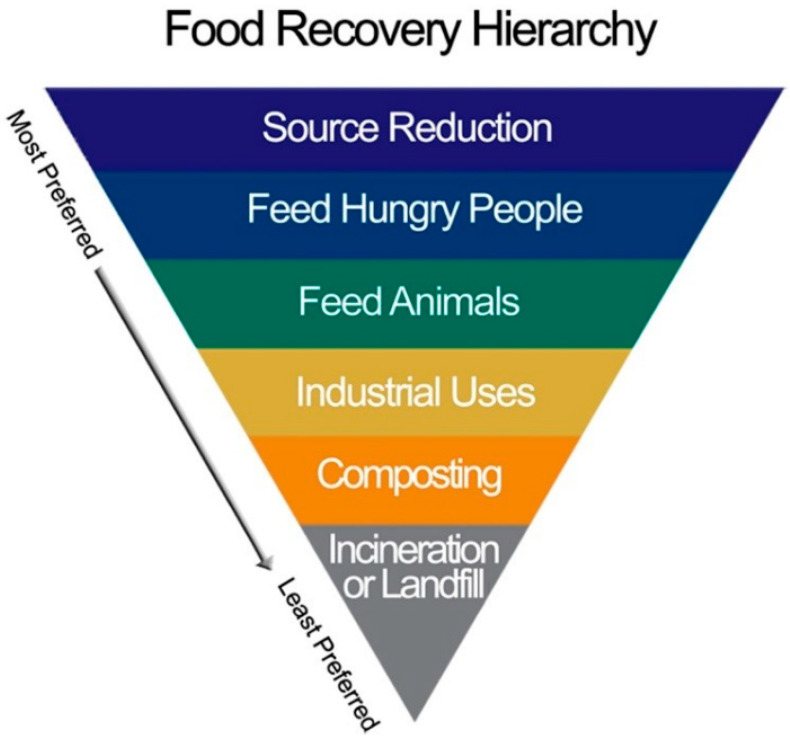
EPA Food Recovery Hierarchy [21].

Benson et al. [31] examined food waste programs and activities at the state and local levels in the United States with information derived from 10 participating public agencies throughout the country at the county and state levels, as well as from one national study. Agency participants often described their food waste prevention and recovery efforts as uncoordinated, with responsibilities being divided between local, county, and state government agencies, and a lack of resources including funding, staff, training, technology, and infrastructure. There was also difficultly in effectively engaging key players throughout the system in support of food waste prevention and recovery. Although food rescue organizations were often the easiest to reach because they were mostly supportive of prevention strategies, they were typically underfunded, understaffed, and under-resourced, making it difficult for potential donors to engage with them. Where connections were made, they often did not last because of high staff turnover at the store or restaurant and volunteers at the food rescue site. Although tax incentives were available as a way to encourage commercial food donation, many businesses were often unaware or did not understand these incentives, or they did not apply to the businesses’ operations. Concerns about food safety liability was given as a major reason why businesses did not donate food. They were unaware of the Bill Emerson Good Samaritan Food Donation Act of 2023, which establishes federal protection from civil and criminal liability for persons involved in the donation and distribution of food and grocery products to individuals in need. The Act extends to those donating the food, including retailers and foodservice operators, institutions of higher learning, and nonprofit organizations receiving the donations, including food banks. Another challenge was the lack of common metrics that could be used to capture the amounts and costs of food waste. For instance, in recovery programs, measurements of food waste were usually made by the donor or food rescue organization by untrained staff, with the donated food being measured on an ad hoc basis, e.g., pounds of recovered food vs. boxes vs. trays or meals. There were rarely attempts to separate or document edible vs. inedible waste or identify what would be sent for recycling rather than composting. There was also perceived conflict between different levels of the EPA Food Recovery Hierarchy (Figure 2; prevention comes first, followed by recovery, diversion to animal feed or industrial uses, composting, and then landfill). Some of these goals were viewed as conflicting or being in direct competition, such as increasing donations vs. promoting better recycling or composting, and there is no credit given for prevention initiatives. Even though prevention is the first and most important option in the hierarchy, it was often given the lowest priority among agencies due to difficulties with implementation; it is just easier to divert to composting. For instance, food rescue volunteers sometimes do not show up at the grocery store to collect edible waste, and store employees then have to dispose of it to costly landfills.

Interviewees described several strategies to build food waste prevention and recovery programs: (1) improving the measurement of waste through metrics including equipment and software installed at individual operations, such as LeanPath, to track food waste in-house by weight and cost and to incentivize employees to waste less as they buy, prepare, and serve food and beverages; (2) creating larger networks with key stakeholders at different levels of government and other organizations that have more of an influence in making policy changes; (3) recognizing the barriers to food donations, including staff shortages or a lack of commitment at food rescue organizations; and (4) broadening education at the national level to show where food waste occurs and to incorporate the Food Recovery Hierarchy into achievable goals and messaging. Specific targeted education food waste campaigns could be initiated in schools and residential homes coupled with websites, media campaigns, and toolkits. On a larger scale, governments, academia, and industries can host food waste conferences, provide technical assistance for food businesses, conduct national or state-wide assessments on food waste, and allocate landfill fees to support food waste reduction.

Barco et al. [32] agree with other researchers that the first step to reduce the current food loss and waste along the agrifood chain with corrective measures is to quantify the generation of food waste, but the methodologies are labor-intensive, as well as inconsistent or incomplete. These authors suggested new ways of automatically identifying potential food wastage generators and agents at the local level. They combined trading income tax, an economic tax which applies to companies carrying out any entrepreneurial or professional activity in a certain territory, with municipal scale points derived from Geographic Information Systems (GISs) to identify food wastage points across the agrifood chain. Trading income tax contains a specific list of different entities with economic activity within in a municipality, as classified by the NACE categories from the European statistical classification of economic activities (Nomenclature of Economic Activities, NACE). This type of information allowed entities that could produce food waste to be identified locally. The authors chose two municipalities in Spain to model the system for its effectiveness. For instance, in one of these municipalities, two economic activities, restaurants and mobile foodservice activities (with 13 entities) and beverage serving activities (with 9 entities) stood out more than the others as operations to target for food wastage measurement and reduction at the local level. From these types of data, the authors argued that it is possible to prioritize studies at the local and regional scales to generate more and better information on food waste at different levels. They also suggested that this methodology would facilitate dialogue between stakeholders about the need to better measure food waste at the local, regional, and national levels for reduction targets in the short, medium, and long term. These adjusted methodologies would help people understand the local issues and generate targeted reduction strategies.

In comparison with other components in the food supply chain, food waste data collection performed in the foodservice sector is more limited. Catering facilities and households are at the very end of the food supply chain, and in Sweden, the public catering sector serves a large number of meals through municipal organizations, including schools, preschools, and elderly care homes. Erickson et al. [33] noted that the first step in identifying where food waste occurs is establishing a baseline measurement. To this aim, they examined Swedish municipal-organized catering establishments, where the amount of food prepared and served was weighed to determine the amount of waste. The results show that there was 64% of serving waste, 33% of plate waste, and 3% of other food waste, with 33–131 g per portion being served (average 75 g). They noted that kindergartens had lower waste than schools, and operations preparing their food in-house had less waste (42%) than facilities receiving warm food prepared in satellite kitchens. The authors speculated that kindergartens had less waste because the carers ate with the children, and in-house kitchens had more control over the amounts prepared and what could be reserved as leftovers. They deduced that waste reduction strategies have to be tailored to specific operations. Aschemann-Witzel et al. [34] discussed the retail display and potential sale of suboptimal food, which is edible but not considered “normal” by consumers through packaging damage, potential spoilage appearance, and closeness to the expiration date, all of which cause these products to be unnecessarily discarded by producers, retailers, and consumers. Specifically, they said that unnecessary food waste occurs at supermarkets, which claim to sell foods that are of higher safety and quality than those found in other retail stores, and they are more concerned about consumers’ perceptions of seeing poor-quality food, any of which, if found, they would choose to discard. However, reduced prices and effective marketing can influence consumers’ reactions to suboptimal food. Available data show that waste arising from foodservice operations, particularly catering, seems to be high in developing countries, in addition to waste emerging from a developing economy that is rapidly moving to a more affluent society. But there is limited information from the many developing countries in different regions of the world, only some of which is covered in the next section.

## 4. How Much Waste Arises from Retail and Foodservice Operations in Developing Countries?

Examples of food waste in three developing countries are discussed below. These have many industrialized segments in food production, similar to those in developed countries, but not throughout the food chain in these countries. The first example is India, as identified through a review by the World Resources Institute India [35]. Despite having undertaken national-level surveys on post-harvest loss, this country has not yet begun reporting on Sustainable Development Goal 12.3 set by the United Nations, and any comparisons between the existing data cannot easily be made because of differences in the measurement metrics chosen. Additionally, studies have not been conducted on the environmental impact of food loss and waste in India. However, what is available at the retail and foodservice levels shows that the issues are somewhat similar to those in developed countries. Defective produce and overproduction were both cited as causes of food waste in the retail, restaurant, and hospitality sectors, mainly in urban centers. A survey of 63 restaurants in Mumbai showed that 75% of the restaurants deliberately overprepare by 10–20%, and some by up to 30%, due to the anticipation of catering to extra customers. This particularly applies to high-end fine dining restaurants compared to other types of restaurants. Food waste has been found to be greater in buffets (22%) than in in-service restaurants (20%), and a large quantity of food is wasted at weddings and social gatherings (10–15%). For instance, 943 metric tons of high-calorie food is wasted at weddings in Bengaluru city every year, which claimed to be enough to serve about 26 million people an average Indian meal. Recommended solutions for reducing wastage are similar to those in developed countries: reducing, reusing, recycling, and composting food waste; donating to food banks; installing a public fridge outside restaurants to provide leftovers to anyone in need; conducting food waste audits of restaurants; allowing clients to choose their serving sizes and to take away leftovers; training staff to minimize wastage; hiring food waste auditors; reducing prices for end stock or offering sales; and selling food to lower-quality shops or for reprocessing into prepared meals. In 2019, the Food Safety and Standards Authority of India (FSSAI), New Delhi, India started enforcing the Recovery and Distribution of Surplus Food Regulations, which specify the responsibilities of food donors and surplus food distribution organizations to donate surplus food freely to any person. The report recommends setting up a multi-stakeholder action coalition to create partnerships to put FLW at the top of the agenda in India and to develop strategies to manage it. This coalition should be able to (1) argue that food loss and waste should be on India’s research agenda; (2) develop specific collaboration and partnerships to reduce FLW; (3) raise awareness of the different dimensions of FLW among diverse stakeholders; (4) develop strategies and mobilize action to reduce FLW, including and the costs and benefits of these proposed actions; and (5) support policy and its implementation for sustainable food systems. However, without strong leadership and stakeholder commitment, including government, these general goals may see little progress. The coalition should be specific in holding the government and the food industry accountable with specific milestones to reach India’s Sustainable Development Goal 12.3. In addition, the foodservice industry should reduce the practice of overpreparing to only 5% or less. It is likely that many of the issues discovered in the World Resources Institute Indian study would apply to many other developing countries where much edible food is simply discarded; incentives should be developed to encourage more food to be donated more efficiently or recycled, especially in regions where there is high poverty.

China is another example of a large developing country that recognizes it has too much food waste. China has 22% of the world’s population but only 7% of the world’s arable land, and food security is a priority for its residents [36]. As the standard of living has increased in that country, consumer food waste also increased, considerably impacting the global food market, resource use, and GHG emissions. Based on the food waste hierarchy [37], an improved food waste disposal system is urgently needed in China. Food waste takes up a high share (ranging from 50% to 70%) in municipal solid waste in Chinese cities, and most food waste is mixed with other municipal solid waste streams and eventually incinerated or landfilled. The Chinese government’s recent crack-down on official extravagance and governmental reception meals at public expenses has shown an immediate impact on restaurant food waste reduction. More bottom-up initiatives from non-governmental organizations and social campaigns, such as the “Clean Your Plate” initiative, should also be encouraged. Other strategies, such as recovering energy via the anaerobic digestion of food waste, recycling food waste via compositing and/or converting it into animal feed, and redistributing food surplus via food banks, should also be explored. According to Li et al. [38], governments, including those in China, need to implement comprehensive legislative requirements to reduce food waste and convert it into useful biproducts, and China has made progress in specifying food waste policies and regulations to promote food waste management, such as source separation, collection, and local treatment. The authors state that the food waste treatment capacity could increase to 40% in China within the decade, but this would require technical guidelines, standards or regulations for food waste management for source separation, pretreatment improvement, and end product utilization, particularly for products such as biogas from anaerobic digestion. However, unlike in some developed countries, where there are financial incentives to support electricity and heat production from renewable food sources, there is currently no government support to do so in China. The authors pay less attention to the prevention of food waste, but they recognize that food waste arises from hospitality and foodservice, food manufacture, the retail and wholesale sectors, and households, as well as from pre- and post-harvest sources, and the authors consider that it is important to raise public awareness about consumers being less extravagant in overordering food and promote keeping leftovers for later. Wang et al. [36] focused more on foodservice, and based on a direct weighing method and a survey of 3557 tables in 195 restaurants in four case cities, they found that the food waste per capita per meal in four cities with different varieties of cuisines was 93 g, which is equivalent to 11 kg/capita/year. This equals approximately 11 kg/capita/year and is not far from that of Western countries, although the per capita GDP of China is still much lower. The breakdown of products wasted was as follows: vegetables (29%), rice (14%), aquatic food (11%), wheat (10%), and pork (8%). Interestingly, they also found that the amount of food waste varies considerably by cities, that tourists waste more than local residents, and that more waste is produced in larger restaurants. Additionally, gatherings of friends and business banquets (serving mainly aquatic products and beef) produce more waste than work gatherings and private dining settings (mainly consumption of wheat products). For instance, consumers in large-sized restaurants wasted, on average, 132 g/capita/meal compared to medium- and small-sized restaurants (77–69 g) and snack bars (38 g/capita/meal). More was wasted per capita per meal after dinners (104 g) compared with after lunches (89 g). The respondents’ awareness of frugal habits significantly related to the amount of food waste, whereas it appeared that awareness on environmental protection, personal health, and food shortage was not an issue. More than 82% of respondents said that they eat the leftover food, 10% fed their pets with the doggie food bags, and 8% eventually discarded it. Most tourists are often uncertain about the quantity and quality of the food when they order, which may consequently result in more waste. In summary, Wang et al. [36] stated that an improved food waste disposal system is urgently needed in China. Food waste takes up a high share in municipal solid waste in Chinese cities, and most food waste is mixed with other municipal solid waste streams and eventually incinerated or landfilled. Other strategies, such as recovering energy through the anaerobic digestion of food waste, recycling food waste by compositing and/or converting it into animal feed, and redistributing food surplus to food banks, should also be explored. Once the government criticized government employee extravagance, especially at publicly funded reception meals, there was an immediate reduction in restaurant food waste, and non-governmental organizations should build on public campaigns such as “Clean Your Plate”. Perhaps it is easier in an authoritarian state than in a democratic one to make changes rapidly, but it also may be more difficult for the ideas of different stakeholders to be heard and acted upon.

The third example is in the Gulf Region where Gulf Cooperation Council (GCC) countries are governed by monarchies. These have experienced growth arising from developing economies to rapid urbanization, with the GCC countries wasting 10 million tons every year [39]. Rising living standards and a lack of awareness were to blame for impulsive buying in relatively affluent GCC countries, causing food waste in this region in ways similar to what occurs in the EU and the United States. One study showed that hungry shoppers spent 60% more and bought more non-food items than less hungry customers, while another survey revealed that those shopping while hungry were likely to buy more high-calorie food items. The hospitality sector contributes significantly to food waste due to overpreparation, buffet excess, and customer plate waste, as it does in other economies. However, there is one difference: the Arabic culture. Big feasts and large amounts of food on the table are directly associated with local generous hospitality, especially during the Islamic holy month of Ramadan, when food wastage almost doubles in the United Arab Emirates (UAE). Another issue is the hot climate, with high temperatures and extended supply chains tending to increase the risk of food spoilage in import-reliant Arabic countries. Because food waste is a significant problem in the UAE, costing the country USD 3.5 billion annually, the UAE launched a national food loss and waste initiative called Ne’ma, which involves encouraging government entities as well as stakeholders from different sectors to cut food loss and waste by 50% by 2030. This is coupled with the UAE’s Food Waste Pledge and Saudi Arabia’s Say Yes to Less campaign. However, as in other countries, it is hard to change local cultures and consumer practices relating to selecting, buying, preparing, and serving food without incentives.

Phonthanukitithaworn et al. [40] made a comparison of policies for reducing food waste in five countries in the EU and five countries in Southeast Asia and found that each country has strategies to reduce food waste nationally, but only the 27 member states (MSs) of the EU have jointly agreed to initiatives, such as the EU Green Deal, which has a comprehensive framework to reduce food waste after the European Parliament called for an enforceable EU-wide food waste reduction target of 50% by 2030. This was based on a common methodology for measuring food waste that directly supports Sustainable Development Goal 12.3. The three main levers of the Green Deal are mainly at the production end of food (reducing chemicals in agriculture, decreasing post-harvest losses, and encouraging more healthy diets for consumers with less animal-based products). Modeling by Phonthanukitithaworn et al. [40] shows that there would be a reduction in GHG emissions and less biodiversity damage, but only at a loss for some producers, particularly those involved with livestock, who would see less feed available for their animals and a lower demand for meat. If such policies were to be implemented in other regions, there would also be winners and losers, and such changes may be difficult to implement, at least in the short term.

## 5. Food Waste Mitigation Strategies

### 5.1. Balancing Sustainability and Food Safety

According to the USDA and EPA, the best approach to reducing food loss and waste is not to create it in the first place. Waste can be avoided by improving product development, storage, shopping/ordering, marketing, labeling, and cooking methods. If excess food is unavoidable, it should be recovered to be donated to hunger relief organizations so that they can feed people in need. Inedible food can be recycled into other products, such as animal feed, compost and worm castings, bioenergy, bioplastics, and clothing, as shown in the Food Recovery Hierarchy (Figure 2). This concept was derived from the “3Rs” concept (reduce, reuse, recycle) and Sustainable Consumption and Production. Sustainable resource management is grounded on the notion that waste can be a resource.

Nevertheless, in policy, it is challenging to balance food waste and food safety. While the reduction of waste in the food chain is clearly an important sustainability issue, some of the seemingly obvious solutions can potentially raise the risk to public health authorities and consumers. Therefore, balancing the desire to decrease food waste and requirements of food safety requires a constant cooperative dialogue to educate both consumers and food entrepreneurs. Kasza et al. [12] specifically raised the issue of safety to receivers of food waste over sustainability to use as much as possible and avoid landfilling. Food waste occurs at all stages in the food production and preparation chain in both low-income and high-income countries, but in low-income countries, food losses including waste appear in the early stages of the food chain, while for high-income countries, more food losses occur in the final stages [41]. It is in these final stages that stakeholders, including processors, retailers, regulators, and potential waste consumers (e.g., food banks and farms for animal feed), have to work together to agree on what and how food waste is acceptable for further use. According to Kasza et al. [12], the key aspects of food safety in relation to food waste are presented in Table 2.

Upcycled food is food that would normally be thrown away or used as animal feed but is instead repurposed to make a new food product [42]. On an industrial scale, some companies use the waste from their own products to make new, upcycled products, while other companies buy and sell upcycled foods. Examples are consumer-unacceptable vegetables that are cosmetically imperfect being combined with hemp seeds to be made into vegan cheeses; pieces of chocolate that are broken during processing, which are converted into chocolate-covered pretzels; and whey that is turned into a clear, vodka-like liquor. Various ways have been proposed to use apple peels; one example is to make apple tea, jelly, syrup, crisps, dog treats, as well as composting [42]. Asioli and Grasso [43] asked 106 consumers in the UK how willing they were to pay (WTP) for biscuits made with upcycled defatted sunflower cake flour. Once they were informed about the nutritional and/or environmental benefits of the upcycled ingredient, the authors saw a significant increase in the WTP for this type of biscuit. Based on these findings, the authors argued that food industries should more seriously consider marketing strategies for upcycled food. However, Rao et al. [44] raised bigger questions about whether certain commodities, such as fruit, vegetable, and meat processing waste, can be safe, sustainable, and nutritionally valuable all at the same time. Obtaining valuable compounds from such waste for use in functional foods may be a way to gain consumer acceptance. Even though the current EU food safety legislation does not allow for food processing by-products, the authors argued that private food safety standards directed to sustainability-related issues in food supply chains may be a way forward for some food processing by-products to be valorized while ensuring sustainability, food safety, and nutritional relevance.

Goodman-Smith et al. [45] made several suggestions for retailers to reduce waste even further: (1) unpackage past expiry date products, particularly meat portions, which can be combined and sent for protein reprocessing; (2) pay more attention to the inventory of short shelf-life items like dairy, meat, and marine products; (3) adopt or improve communication strategies between retailers and consumers about restocking practices; (4) inform consumers that misshapen fruits and vegetables are normal and should not be ignored; (5) reduce sales of bagged salads that spoil quickly at home; (6) explore ways to donate close-to-expiry dairy products to charities with refrigeration; (7) phase out BOGO sales (buy one, get one free) as this encourages consumer overpurchase and increases waste; (8) stress the environmental concerns of retail and domestic food waste; (9) continue to help purchasers understand the different dating systems retailers use; and (10) understand how the no-liability clause exonerates retailers who donate to charities provided they can show that the donations were considered safe at the time of donation (equivalent to the Good Samaritan Act [30]).

Retailers need to understand and measure consumers’ awareness and concerns about food waste to impact their purchasing patterns and consider educational strategies to prevent unnecessary waste.

### 5.2. Economic Incentives

Economic incentives aim to reduce food waste through costs or other market signals [46,47]. They can be categorized into fees, taxes, and subsidies. Charging households for personally generated waste has been found to be an effective scheme to reduce food waste. In contrast, shoppers who consider themselves frugal and would be inclined to purchase and eat suboptimal food would contribute to minimizing waste [12]. The authors note that shoppers build a relationship with retailers by noticing the practices employees carry out and how they are treated by staff. Displays of suboptimal food for sale are negatively interpreted by shoppers, but they can be influenced by price drops and better communication on what suboptimal means. The authors recommend conducting further research into building consumer trust to increase understanding about suboptimal food and avoid unnecessary waste. Retailers and producers in New Zealand (NZ) prefer sending food waste to farms rather than to landfills [45]. However, this practice is less used in European countries because of the risk of spreading diseases to animals, such as African swine fever and foot and mouth disease, and in fact, it is illegal unless the waste has been temperature-treated. More specifically, Goodman-Smith et al. [45] found that fresh vegetables and fruits and baked items were the most products to be binned by retailers in NZ by weight (44% and 23% of the total food wasted and diverted, respectively), but the amount of fruits and vegetables that were discarded was even higher in Austria (68%), mostly because of perceived imperfections by shoppers. To counteract this, the NZ government encourages retailers to transport food waste away from landfills and give it to local farmers and food rescue charities or use it for protein reprocessing. New Zealand also has a “Good Samaritan” policy to allow businesses to donate to food banks or food rescue groups without penalty, provided that the food is safe at the time of donation. France and other European countries have even more extreme policies that penalize large supermarkets if they discard unsold food, forcing them to donate this food to food banks or other charities. However, there are concerns about this type of policy, if subsidized, that would perpetuate a low standard of living and poverty in these countries with the use of public funds. In NZ, there is a public preference for the industry to create its own initiatives without government interference. Goodman-Smith et al. [45] found that in NZ, the retail industry is more inspired to reduce food waste by being environmentally conscious not to use landfills, having pride and satisfaction by donating food to vulnerable members of the community, obtaining economical gains by using the more costly landfill option less often, and doing the “right” thing by being socially responsible in the community. However, these strengths are impeded by a lack of clarity and purpose for employees to understand the importance of a policy of having less food waste, which requires more employee training and education; worry about causing animals or people to become sick after eating the donated expired food; and customer perceptions of quality *(“the reason why we throw it away is because if we don’t throw it away, they won’t buy any of our produce, it’s a vicious circle”*). Retailers also have to donate what farmers and charities want, and dairy, meat, and fish items are typically not accepted; limited staff resources means it is easier for food to be diverted to landfills than to apply the logistics of donations. However, overall, the retail industry is performing better than households (3 kg/capita/year versus 29 kg/capita/year sent to landfills by the retail sector and homes, respectively), with about 77% of all retail food waste avoiding landfills; about 15% of all waste is donated to charities, and 50% waste is sent to farms as animal feed, mainly for pigs and chickens (6 kg/capita/year). However, to avoid the spread of pathogens to farm animals, any food waste that has come into contact with meat must be heat-treated. Unfortunately, much of the food that is assigned to feed animals is known to be perfectly safe for human consumption, and if the parties involved could be convinced, it could be donated to food banks or equivalent charities. The United States has similar proportions of retail food waste that has been diverted from going to landfills (72%).

**Figure 3:**
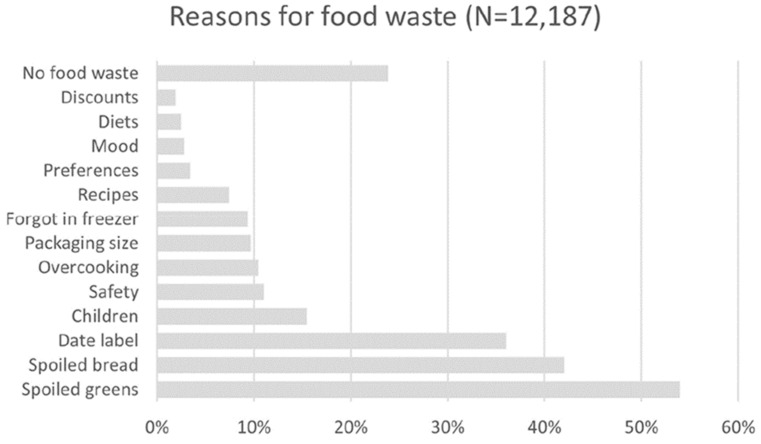
Reasons given for food waste according to the study by Närvänen et al. [48]. Reproduced with open access from Wiley.

Närvänen et al. [48] argued that retailers can increase efforts to advise purchasers, specifically in the “Trust date labels” and “Food safety first” segments, regarding their understanding of different types of date labeling and what spoilage appearances look like to make safe decisions. However, these types of customers may not want food that is near its expiration date, unlike “Occasional wasters” or “Overpurchasers and overpreparers”. However, this segment might appreciate apps that provide advice on refrigerator storage practices and suggest recipes for quick meals. The UK temperature-sensitive Mimica touch is a cap or label that helps consumers store their food properly and to know when food is truly spoiled to avoid undue waste. It essentially comprises a bumpy plastic base, a gel in the middle, and a film layer on top. This “bio-responsive” gel reacts at the same rate that food spoils [49]. When it liquifies, bumps are visible, and they can be felt to indicate that the food is spoiled. It is particularly designed to recognize when the package is opened, causing the food to be exposed to the air, which enhances the spoilage process. It is currently in use on juice bottles, but meat is the next food to be considered (as well as vaccines). The authors conclude that retail managers should be responsible corporate leaders and encourage customers to utilize loyalty programs to help their customers understand how to reduce food waste globally. However, Närvänen et al. [48] pointed out that their data are based on Finnish studies, and the conclusions may not be pertinent to consumer segments in other communities (Figure 3). We should also add that this survey study equated the rejection of spoiled greens and bread with concerns about food safety, but they are not directly linked; pathogens may multiply in foods that do not easily spoil, and many of these foods may spoil without the presence of any pathogens. In fact, these types of products, including berries and breads, are not typical vehicles for foodborne pathogens.

Since about 6% of the products delivered to supermarkets is binned, increasingly, large retail stores are using computer-assisted ordering or automated ordering systems to determine when stock, typically non-perishable items, need to be replenished. These systems can also determine the age of existing inventory, thus avoiding large periodic orders to replace low inventories with the likelihood that the stock will expire on the same date and be unsellable [50]. For instance, smart packaging with RFID tags can be used to keep track of time and the temperature to which products are exposed when moving through the supply chain, as well as to measure a change in the gas composition inside the package, which can help to predict food quality and ensure food safety. Food waste can be reduced by lowering the temperature at supermarkets to extend shelf life, particularly for meat and dairy products; this may positively impact retailer costs and reduce greenhouse gas emissions. Similar to retail shoppers, foodservice patrons expect to have a variety of good-quality food on the menu, and once the order is made, the food is expected to be served quickly. This allows for certain redundancy in having enough available food through overordering ingredients or subcontracting whole meals, together with waste through improper storage and discards through meal preparation. This is on top of the plate waste from customers who do not eat what was served or selected from buffet lines, accounting for up to 70% of the total food waste at the foodservice level.

### 5.3. Community-Led Solutions to Tackle Food Waste

Governments can also help reduce food waste through economic incentives or penalties, such as exacting fees for waste disposal, but also relaxing unnecessarily strict food safety standards for when food can no longer be sold, e.g., extending or eliminating best before dates [51]. Chalak et al. [52] believe that the elimination of unnecessary food safety standards would be more effective in reducing food waste than introducing economic incentives. In 2012, the European Commission initiated a project to measure, monitor, and eventually reduce wasteful food items called Food Use for Social Innovation by Optimizing Waste Prevention Strategies, or FUSIONS [19]. Because food waste flow tends to combine domestic and business sources, these authors measured urban food waste (UFW) from foodservice facilities, shops, and homes. These authors identified various trends among such urban populations, largely based on Italian data. Some demographic groups are more likely to discard fewer food items (as indicated by Närvänen et al. [48]). For instance, older people in general tend to consume less food; immigrants to the community believe they are critically observed by long-term residents for societal correctness, including limiting waste disposal, and women are more likely than men to discard food less or recycle items more because they are more attuned to environmental care. In contrast, households with children are more likely to have wasteful practices because children’s eating habits are unpredictable, with food often being left on the plate, whether from meals or snacks; those who eat out frequently and spend money are less likely to feel guilty about wasting food at restaurants or letting food spoil at home; an active social life can lead to unplanned dinner events with friends, resulting in earlier store-bought food remaining uneaten and then being discarded; and families who host events that prepare, or have catered, freshly prepared meals and do not serve leftovers from previous meals will generate more leftovers, all of which will likely be discarded in the near future.

Leftovers may also be unnecessarily discarded because such food is considered boring to eat a second or third time, is of lower quality, or considered too risky to serve to family or friends. In contrast, some home preparers are more frugal about wasting food unnecessarily and will be more aware of safe storage times, using their lifetime experience and their accumulated food safety knowledge. Unemployed individuals are also more likely to be frugal since they have fewer opportunities for food choices and will make economic decisions on what to keep and what to discard. When food is to be discarded, food waste may first be given to pets rather than being dumped into the garbage straight away. Watson and Meah [53] collected comments on waste; one individual mentioned the following: “*So in an ideal world I wouldn’t waste anything but, I am aware that I probably do waste things because I’m trying to, because it’s part of the compromise*”. They quoted previous work that says “food waste emerges from the intersection of ‘time, tastes, conventions, family relations and domestic divisions of labour” within “the material context … of domestic technologies, infrastructures of provision and the materiality properties of food itself”. The authors concluded that media campaigns focusing on consumers feeling more responsible for preserving the environment are limited in their impact to reduce food waste, but thrift ethics seem to be bigger drivers in determining what becomes discarded and what is not. They state that thrift towards food acquisition and storage is partly determined by the relative costs of income and time. In contrast, food safety may not be a major issue for households in which family history and culture may be more important when deciding what should be discarded.

More equitable distribution of food is being encouraged in many European countries as well as in the US. For example, in the United Kingdom (UK), there has been a campaign for over 15 years called “Love Food Hate Waste” with advice on shopping tips, meal planning, and portion sizes, along with advice on how to store different types of foods (clarifying use-by and best before dates) and how to serve leftover items [53]. Based on the UK campaign, the similar New Zealand government’s three-year program changed some attitudes to increase awareness of the value of food and advise people not to waste so much [45]. Cerciello et al. [19] provided other examples. In Poland, food sharing places like “Jadłodzielnie” are sites where food surpluses are stored in refrigerators from which everyone can take something for themselves or leave it for others, a nationwide initiative aimed at reducing food waste [54]. This initiative is less of a supply for individuals in need and more about building awareness in the population about the need to reduce food waste without burdensome administrative oversight. Food sharing is coordinated by a group of volunteers who build connections with local supermarkets and bakeries to facilitate the sharing of food that would otherwise be wasted. Starting as a protest against fast food and oppressive food systems in Italy in the 1980s, “Slow Food” has become a million-member movement of food activists around the world [55]. In the United States, Slow Food partners with other organizations, trying to inform and aid large-scale producers (including farms, processors, local governments, and retailers) on how to “reduce, reuse and recover” their food waste and distribute produce to families who rely on food assistance. “Slow Fish” engages activists, fishers, shrimpers, chefs, and consumers who are faced with making choices about seafood every day and encourages building a sustainable future together. One particular issue is stopping wasteful bycatch. “SHARECITY” is an international research project that explores food sharing in cities across the world, and it began in 2016 [56]. The project grew into a large database of food sharing initiatives, including everything from collective growing groups to community cooking events. The concept relies on an open-source map known as “SHARECITY100”, which maps over 4000 initiatives in 100 cities in 44 countries and six continents. Examples of these initiatives are community gardens, eating together in community kitchens, or participating in surplus food redistribution. The founders claim that “food sharing initiatives have been acting ahead of the policy curve on matters of food waste reduction”, but “there is still much to be done by all actors… food businesses and consumers that create the waste in the first place are also key, as are policymakers who can support people to reduce waste in many ways”.

The United States Environmental Protection Agency (EPA) aims to reduce food waste to 164 pounds per person by 2030. Of the food wasted, 42% consists of fresh fruits and vegetables, while 26% comprises milk and dairy products. A mere 15% reduction in food waste could not only combat climate change but also provide enough food to feed over 25 million Americans annually. Food Rescue US (FRUS)-Lansing Communities (Lansing, MI, USA) is one of many Food Rescue US operations nationwide. This one actively addresses this issue by rescuing perishable food from various donors, including grocery stores, schools, bakeries, and farmers, and delivering it directly to social service agencies that serve hungry individuals in Lansing. Utilizing an app, volunteers claim rescues and coordinate timely deliveries, with the entire process taking approximately 30–40 min. Despite these efforts, research indicates that less than 6% of the wasted food is donated, while the majority ends up in landfills. With the ongoing increase in demand for food assistance, particularly in the wake of the COVID-19 pandemic, Food Rescue US is one of many volunteer organizations playing a vital role in combating food waste and hunger in the United States [13,24].

### 5.4. Innovative Strategies to Reduce Waste

Even though retailers and foodservice operators are the source of food that is often wasted, consumers have to be involved in the overall strategy of waste prevention. About 42% of food waste in Europe takes place in people’s households, 60% of which can be avoided by better practices, according to the FAO [57]. In Spain, 1.43 million tons of food is thrown in the trash annually, with fruits, bread, and vegetables being the household items most wasted. In Spain, 8 million tons of food is wasted each year, making it the seventh highest food waster in the EU. The “Yo No Desperdicio” [I Don’t Waste] website and mobile app has been operational since 2016 to “to promote alternative ways of responsible and sustainable consumption, and to make us rethink our role as consumers, with the goal of reducing food waste at home”. “*The platform offers a very simple food-sharing method. Users log into the website or app and post an ad describing the food they want to offer, complete with the item’s name, picture capturing its current state, portion, location and an expiration date. Both parties then arrange for the free delivery of the products*”. On this food sharing app, users post a picture of the item they are offering with information such as the quantity, location, and expiration date. Both parties arrange the swap of their items in a private message. Members also share recipes and tips for preventing food waste. However, a little more than 100 pounds of food was exchanged in the first year (including dairy, fruits and vegetables, sodas, dried fruit, eggs, and canned food), which is a very modest amount compared with how much food is discarded by households every year, because users have been slow to trust the platform, as indicated by Cerciello et al. [19].

Bozhinova [58] listed 14 mobile apps devoted to reducing food waste, including Flash Food in Canada and the United States. This app alerts potential buyers about grocery foods approaching their best before dates and encourages them to sell the foods at a discount. This approach is similar to “No FoodWasted”, which aims to reduce food waste in the Netherlands by 50% in the next five years with an app for discounted products with best before dates. These apps remove potential wasted food from retailers’ inventories, but it is probable that some of this food will be wasted by consumers because of the short best before period remaining. “Food for All” connects customers to restaurants in Boston and New York City one hour before they close for meal discounts as high as 80%. Food Cowboy arranges efficient communication between food donors and charities and allows for the fast delivery of excess food in the United States. Delivery drivers, caterers, and anyone working with large volumes of edible but rejected food create alerts in the app. Food pantries, processors, and composters immediately receive these alerts and contact the source for delivery arrangements. Apeel Sciences, a food waste technology company, acquired the hyperspectral imaging startup ImpactVision, which uses advanced imaging technology to assess food for quality and safety. The company has deployed its Fruitcam in packing houses and distribution centers across the Americas and Europe to assess the quality of fruits and vegetables, so wholesalers can decide which ones to ship to long distances and which to sell locally, and their Fishcam can mark the difference between fresh and frozen fish filets. ImpactVision enables fast decision making based on food quality. “No Food Waste” crowd-sources data on hunger spots in India to facilitate surplus food donations, with 80 locations in Delhi and the capital region. Users themselves can mark hunger spots, which the team verifies and enters into its database. Users can also donate food or request the app to deliver the donation using its volunteer drivers. So far, No Food Waste has fed 500,704 people, saving 165 tons of food waste. A unique solution for disposing of unwanted carcass meats has been explored in Europe where vultures are present. These birds are rare in Southern Europe and, unfortunately, their habits of feeding in large groups render these birds vulnerable to mass poisoning if the carcasses they eat are contaminated. Therefore, in recent decades, feeding stations have significantly aided in the recovery of vulture populations across Europe. In Cyprus, four state-managed restaurants operate within vulture territories, with meat scraps providing sufficient and safe food for these birds [59]. Also, controlled feeding areas near farms are being explored where, in collaboration with farmers and veterinary services, carcasses will be placed for vultures instead of being transported to incineration facilities.

Moreover, raising awareness about the environmental, social, and economic impacts of food waste encourages consumers to reconsider their consumption habits, but it is necessary for industry, NGOs, and government stakeholders to promote national and international awareness programs. The Zero Food Waste Coalition proposes two key actions to reduce consumer food waste: funding research and implementing awareness campaigns by drawing from successful initiatives in countries like the UK and the Republic of Korea. The School Food Recovery Act, introduced in congress, seeks to enlist schools in food loss and waste efforts by providing funding for waste measurement and reduction initiatives. Programs like the World Wildlife Fund (WWF)’s “Food Waste Warrior” and the Natural Resources Defense Council (NRDC)’s “True Food, No Waste” have shown promise in decreasing student plate waste and plastic packaging while increasing fruit and vegetable consumption [60]. In January of 2020, the NRDC, WWF, Harvard Food Law Policy Clinic, and ReFED launched an informal coalition to engage with and inform policymakers on opportunities to prevent and reduce food loss and waste. Together, they developed and published the US Food Waste Action Plan to reduce food waste by 50% by 2030, and it is endorsed by over 60 businesses, nonprofits, and local governments.

These various initiatives, spanning from food redistribution programs, technological advancements in food preservation and distribution, and policies promoting sustainable agriculture practices, all play crucial roles in tackling this global issue. Moreover, it is imperative to foster balanced views between considerations of public health, economic viability, and food safety when implementing strategies to combat food waste.

## 6. Conclusions

Increasingly, governments and the food industry are recognizing that across different regions, significant amounts of food are being discarded at various stages in the supply chain. Retailers and consumers want to take action to prevent undue waste, but reducing waste is particularly challenging because of many conflicting factors such as perceived food safety risks, economic considerations, and retailers’ marketing strategies and inventory management, which all play crucial roles in determining how much food waste is generated and disposed of. These multifaceted causes require a comprehensive approach involving various stakeholders, including governments, businesses, and communities, to focus on consumer education and awareness campaigns that promote responsible consumption practices, proper food storage, and the utilization of leftovers. The retail and foodservice industries have pivotal roles in working with suppliers (farmers, producers, wholesalers, and transporters) and users (consumers) to provide enough food items for sufficient choice and quantity, but not to overestimate their needs. To this end, collaborative efforts, informed by successful initiatives from around the world, demonstrate that there is potential to make substantial progress in reducing food waste. However, adopting a balanced approach that considers public health and food safety concerns must be central in tackling food waste across different scales, specifically at the consumer level. Some of the challenges are discussed below.

Many of the surveys show that the solutions to reducing food waste are similar, but before any further studies are conducted, there should be clear definitions of food loss and food waste and whether or not these can be combined (FLW), and this is one of the issues that food chain stakeholders, NGOs, and governments have to address before tackling metrics. At the retail and foodservice (hospitality) levels, discarded food is considered food waste, and it should be separated into edible and non-edible waste, but this is not always carried out. Additionally, there are metrics that are more easily measurable than others, and the focus should start with these (type and weight); several countries, including those in the EU, have a reporting program that can be made mandatory by MSs, but there are no penalties to date for avoiding these. However, in the UK, there is pressure to make it nationally mandatory for businesses to self-report both edible and non-edible waste [61]. The process of achieving this seems to be very convoluted so far as it has not yet been implemented despite considerable enthusiasm being shown by all stakeholders. The WRAP (Waste & Resources Action Programme, which is a charity that works with businesses, individuals, and communities to achieve a circular economy by helping them reduce waste, develop sustainable products, and use resources in an efficient way), the Climate Change Committee and the House of Commons Environment, and the Food and Rural Affairs Committee all agreed for several years that it was overdue to mandate food waste to be reported if they wanted to achieve the UN’s Sustainable Development Goal 12.3 to halve global food waste by 2030, and that this mandate should be implemented immediately. It received support from elected members of parliament (MPs) across party lines to introduce food waste reporting across all sectors, including primary production, manufacturing, retail, and hospitality and foodservice. Over 16,500 people signed this Make Food Waste Count petition urging the government to make it a legal requirement for all large- and medium-sized businesses to measure and report their food waste by 2024. Most businesses are in favor of this, including 67% of primary producers, 79% of retailers, 73% of hospitality providers, as well as industry and environment associations. The government’s impact assessment found that food waste measurement at a business level would only cost 19.18 EUR/tonne of food waste targeted, which is considered a good value, and businesses would see savings of EUR 1200 to EUR 3100 per tonne of food waste. It was calculated that if mandatory food waste reporting in England led to just a 1% reduction in food waste, this would result in net savings of an estimated EUR 24.4 million per year for food businesses. It was also claimed that the land freed up from reducing UK food waste being sent to landfills by 50% from farm to fork could produce enough potatoes and peas to feed 28% of the UK population. However, in mid-2023, the Department of Environment, Food & Rural Affairs (DEFRA) claimed that the cost of imposing mandatory reporting on businesses could drive up food prices even though there was overwhelming support for mandatory reporting in its official consultation. In late 2023, there was a cabinet reshuffle, and the new DEFRA secretary went back on DEFRA’s decision to abandon plans to introduce mandatory food waste reporting by promising that he would “reconsider” the measures. DEFRA has now conducted a survey targeted at large businesses only, and it will look at how the “potential financial costs or savings associated with measuring and reporting would impact consumers”. This could still delay tactics to actually initiate the policy, especially with the upcoming election in July 2024, and there are also no clear directions yet on how mandatory reporting would be administered with government oversight. This is an example of a promise without total commitment because of other priorities or lobbying to a proposal that most stakeholders want to implement soon.

In contrast, the United States has a plan, if not a commitment. A model ordinance was created by the NRDC and the Environmental Law Institute, which can be used by municipalities to require businesses, universities, and other large organizations to report the amounts of food waste and surplus food they generate [62]. This requires a qualified business to document the amount of food waste generated during the prior year by weight, the amount by standard units, and the types of surplus food donated to a nonprofit organization. They should also document major donation challenges that need to be overcome, such as donation logistics, storage, and transportation. Also, they should record major food scrap recycling challenges that had to be overcome, such as odor, staff training, or the availability of organics recyclers. Companies are also allowed to employ approximation methods to generate weight estimates. To help start the process, municipalities are to provide educational tools to train businesses that qualify and be prepared to prosecute any entity that violates these requirements. We note that this is only a model, and it has not been adopted by any municipality to date, but it can quickly be put in place if elected officials make this their policy.

Perhaps mandatory waste reporting can be initiated in stages where certain parts of the supply food chain can more easily be targeted to obtain baseline data to know how and where the key elements of waste occur. Barco et al. [32] showed that restaurants, mobile foodservice activities, and beverage-serving operations were more likely than other operations to generate food waste at the local level (based on two Spanish communities only). One downside of these operations recording their waste is that some of them are small operations that would not have the staff or funds to collect such data. And the same may apply to many operations in the food supply chain in developing countries.

Producing less food waste (expanding the concept of reduce, reuse, recycle into a food recovery/sustainability hierarchy) is like setting targets and measures to reduce GHG emissions; small gains are very slow and painful to achieve, and continuing efforts to reduce waste may be seen as counterproductive in the face of other priorities. A long-term strategy for reducing food waste may be too great a commitment on national agendas for major policy changes no matter what national and international policy think-tanks wish for and recommend. The public sets the priorities in democratic countries, and actions that are too difficult, too expensive, and too esoteric will not receive the momentum to force change unless champions step forward to use social media, the press, and political lobbying to alert the voting public to ask for meaningful policies that the public can see are effective. Several reviews show that a broad coalition of stakeholders would have a bigger impact on reducing waste if they are willing to work together and have recommendations that could be acted upon. But there is little evidence that this works at national or international levels. Those who provide food (retail and foodservice) have to somehow integrate supply chain information with the less certain information from consumers at the purchasing side where attitudes, customs, and behaviors play important roles.

We hope we addressed the issue that achieving an apparently simple goal of limiting food waste across all food sectors, but particularly in the retail and hospitality sectors, is very complex because of all the stakeholders involved, some who have competing interests. Although this review does not attempt to be all-encompassing in addressing the existing and potential policies globally, some progress is being made to meet Sustainable Development Goal 12.3 set by the United Nations to achieve a 50% reduction in food waste by 2030. This may be an ideal goal to aim for, but in the six years remaining, there may be less commitment or resources in the government and industry, in addition to feasibility barriers, to achieve this goal.

## Figures and Tables

**Table 1:** The causes of food waste at the retail level and reduction practices [9], with permission from Elsevier, Amsterdam, The Netherlands, 2020.

Food Waste Reduction Practices	Food Waste Causes
Collaboration	Lack of information sharing Poor inventory control/management policy/lack of stock rotation Inappropriate work procedures Lack of coordination/collaboration
Adoption of food donation practices	Inappropriate work procedures
Price and promotion policies	Standards of appearance and shape Lack of operational control Lack of integrated IT systems Poor inventory control/management policy/lack of stock rotation
Lean supply practices/sequencing	Lack of operational control
Communication with supply chain members	Lack of information sharing Lack of coordination/collaboration
Traceability (in both transportation and supply chains)	Lack of integrated IT systems Lack of information sharing Poor inventory control/management policy/lack of stock rotation
Inventory policy	Poor inventory control/management policy/lack of stock rotation
Secondary channels/use of surplus by other links	Standards of appearance and shape Lack of information sharing
Coordination mechanisms	Lack of coordination/collaboration
Flexibility in quality standards	Standards of appearance and shape Lack of information sharing
Waste reduction-oriented operational systems	Lack of integrated IT systems Lack of information sharing Poor inventory control/management policy/lack of stock rotation
Management autonomy	Standards of appearance and shape
Technology and sensors for food quality control	Problems with transport equipment Problems with display Lack of refrigerated transport Problems with storage Cold chain breaking
Application of thermal control in packaging and/or facilities	Problems with storage Cold chain breaking
Training for waste reduction/prevention	Lack of training
Employee awareness of waste	Incorrect handling Lack of knowledge Lack of training
More accurate labeling information (expiry dates)	Short shelf life
Packaging development and optimization	Inadequate packaging
Law flexibility without compromising consumer health	Very restrictive laws
More precise demand forecast	Inadequate demand forecasting Excess production
Demand management/history technology	Inadequate demand forecasting Excess production Sudden changes in orders

**Table 2:** Food waste management methods and related food safety risks. Reproduced from Kasza et al. [12] with permission from Elsevier, Amsterdam, The Netherlands, 2019.

Priority Grade	Food Waste Management	Methods	Food Safety Risks	Management of Risks
1	Prevention	The forecasting of demand, planning of resources and manufacturing, coordination of distribution, and planning of shopping.	No risks.	No risks.
2	Donation	Edible food stuffs are donated to poor people both from companies and households. Tax deduction could be a good incentive.	Food safety rules and expiry dates have to be respected. Illegal trafficking (e.g., re-labeling or via catering) should be prevented.	The organization of donations is needed for effective distribution. Organizations must be prepared in the field of food safety, and food safety authorities should take part if necessary.
3	Valorization	Animal feed, industrial recycling, and composting.	Elevated food safety risks: illegal trafficking and zoonotic diseases.	Materials suitable for valorization have to be categorized, and material flow should be controlled. The basic rules of risk prevention have to be explained to the broad public.
4	Waste treatment	The safe disposal of waste to landfills or incineration. This stage has to be avoided unless special conditions (e.g., contamination with a highly contagious pathogen) are present	Foodstuffs at this stage must be prevented from being reintroduced to the food chain.	The control of the waste management process and facilities.

## Data Availability

No new data were created or analyzed in this study. Data sharing is not applicable to this article.
